# Case of Parotid Fistula, Cured by the Concentrated Sulphuric Acid

**Published:** 1830-01

**Authors:** John Higginbottom


### PAROTID FISTULA.

*Case of Parotid Fistula, cured by the Concentrated Sulphuric Acid.* By John Higginbottom, M.R.C.S.L.

Miss Brooks, aged seventeen years, received, by instruments used at the time of her birth, a severe injury on the right side of the head and face. Several abscesses formed, which were opened, and some exfoliation took place above the angle of the lower maxilla. Two of the openings made with the lancet never healed, but terminated in salivary fistula; one being situated in the cavity between the mastoid process of the temporal bone, and the condyloid process of the jaw; and the other a little anteriorly to the ear, immediately below the zygoma. The saliva flowed so freely from the openings near the mastoid process, that the patient's neck was constantly in a state of excoriation, and it was necessary to wear napkins constantly upon the breast. Sometimes the orifices would close alternately, but, when that was the case, there was a double discharge from the open one. There was, of course, an increased discharge during mastication.

I first wished to try the most simple means, and attempted to form an adherent eschar by the nitrate of silver over each orifice. This plan succeeded in healing that orifice which was situated anteriorly to the ear, on the first application, but it failed in the other: the eschar remained adherent only for a few days, when it was thrown off, and an increased flow of saliva followed.

When this plan had failed, I used the nitrate of silver, and afterwards as firm pressure as the situation would admit, applied by means of orange peas, plates of lead, and adhesive plaster.

This plan having been continued for a long time without any advantage, I had at last recourse to filling the little cavity with the concentrated sulphuric acid, by means of a feather, every fifth day. I soon found that there was no discharge between the times of applying the acid; but, on delaying its application for a few days, the discharge returned. I therefore continued to apply the acid every fifth day, for eight or ten times. At length, on discontinuing it, I found there was no return of the flow of saliva.

This fistula has now been perfectly cured for nearly three years.

